# El odontólogo y la comunidad escolar

**DOI:** 10.21142/2523-2754-1001-2022-092

**Published:** 2022-03-30

**Authors:** Guido Alberto Perona Miguel de Priego

**Affiliations:** 1 División de Odontopediatría, Universidad Científica del Sur, Lima, Perú. guidoperona54@gmail.com Universidad Científica del Sur División de Odontopediatría Universidad Científica del Sur Lima Peru guidoperona54@gmail.com

El sábado 5 de marzo del presente año, el diario oficial *El Peruano* publicó la Ley N.^o^ 31431, Ley que Incorpora al Profesional Odontólogo en la Comunidad Educativa.

Esta norma, en su Artículo 1. Objeto de la presente ley, señala que “La presente ley tiene por objeto incorporar al profesional odontólogo como miembro integrante de la Comunidad Educativa establecida en la Ley 28044, Ley General de Educación, con el fin de contribuir en la promoción de la higiene y salud bucal para la prevención de enfermedades bucodentales” ^1^.

Asimismo, en su Artículo 52. Conformación y participación, especifica lo siguiente: “La comunidad educativa está conformada por estudiantes, padres de familia, profesores, directivos, administrativos, profesional en psicología, profesional en enfermería, profesional odontólogo, exalumnos y miembros de la comunidad local. Según las características de la Institución Educativa, sus representantes integran el Consejo Educativo Institucional y participan en la formulación y ejecución del Proyecto Educativo en lo que respectivamente les corresponda. La participación de los integrantes de la comunidad educativa se realiza mediante formas democráticas de asociación, a través de la elección libre, universal y secreta de sus representantes” ^1^.

Dentro de los noventa días de promulgada esta ley, se publicará el reglamento.

Con esta ley, las autoridades pertinentes, así como las instituciones académicas, científicas y gremiales, deben organizarse para difundir la Educación para la Salud Bucodental, mediante la Educación para la Salud (EpS) como función social de la comunidad y para el desarrollo de una sociedad justa. La EpS interviene en diferentes campos de acción, como la familia, la escuela, el trabajo, el consultorio de la posta médica o centro de salud, y las instituciones académicas, como las universidades en sus cursos de odontología social o comunitaria ^2^^-^^5^. Riquelme manifiesta que “la educación para la salud es una poderosa herramienta para el quehacer profesional en Atención Primaria, ya que dentro de los servicios de salud es el que ocupa el lugar más cercano a la ciudadanía. Se trata de un instrumento que sirve tanto para la cura y la rehabilitación como para la prevención y la promoción de la salud” ^4^.

Con el retorno a la presencialidad y el cumplimiento de las normas de bioseguridad, los centros educativos se convierten en los lugares ideales para desarrollar la EpS bucodental y acercarla al alumno, la familia y, sobre todo, los miembros de la comunidad escolar. Esta intervención debe iniciarse desde los primeros niveles de educación, como nido, jardín, primaria y llegar en forma secuencial hasta la secundaria con los adolescentes, para lograr comportamientos saludables acompañados de creencias y buenas aptitudes ^6^.

Está demostrado que cuando más temprano se de esta información a los alumnos, más rápido se establecen los hábitos de buena salud bucal y esto genera un mayor impacto de salud bucodental en la sociedad. Toda esta información se debe mantener en forma permanente para reforzarla a lo largo de los años escolares. 

Con la presencia del odontólogo en la escuela, se pueden promover las loncheras saludables, los cuidados y recomendaciones sobre trauma dental y, si es posible, la instalación de un consultorio dental en el que se brinde atención dental oportuna en el ambiente escolar, de modo que el alumno se convierta en un agente vivo de la salud bucodental en la familia. Esta es una buena oportunidad que se le presenta a la odontología peruana ^7^.
